# Analysis of GWAS-nominated loci for lung cancer and COPD revealed a new asthma locus

**DOI:** 10.1186/s12890-022-01890-7

**Published:** 2022-04-23

**Authors:** Anne-Marie Madore, Yohan Bossé, Patricia Margaritte-Jeannin, Emily Vucic, Wan L. Lam, Emmanuelle Bouzigon, Jean Bourbeau, Catherine Laprise

**Affiliations:** 1 Département des Sciences fondamentales, Université du Québec à Chicoutimi, Saguenay, QC G7H 2B1 Canada; 2 Institut universitaire de cardiologie et de pneumologie de Québec – Université Laval, Quebec, QC G1V 4G5 Canada; 3 Department of Molecular Medicine, Université Laval, Quebec, QC G1V 0A6 Canada; 4 UMRS 1124, INSERM, Université de Paris, 75006 Paris, France; 5 Department of Biochemistry and Molecular Pharmacology, New York University School of Medicine, New York, NY 10016 USA; 6 Department of Integrative Oncology, British Columbia Cancer Research Centre, Vancouver, BC V5Z 1L3 Canada; 7 Canadian Environmental Exposures in Cancer (CE2C) Network (CE2C.Ca), Halifax, Canada; 8 Research Institute of the McGill University Health Centre, McGill University, Montreal, QC H3H 2R9 Canada; 9 Centre intersectoriel en santé durable, Université du Québec à Chicoutimi, Saguenay, QC G7H 2B1 Canada; 10 Canada Research Chair on Environment and Genetics of Respiratory Diseases and Allergy, Université du Québec à Chicoutimi, Saguenay, QC G7H 2B1 Canada

**Keywords:** Asthma, COPD, Lung cancer, Genetics, 2p24.3, *MYCN*

## Abstract

**Background:**

Asthma, lung cancer (LC) and chronic obstructive pulmonary disease (COPD) are three respiratory diseases characterized by complex mechanisms underlying and genetic predispositions, with asthma having the highest calculated heritability. Despite efforts deployed in the last decades, only a small part of its heritability has been elucidated. It was hypothesized that shared genetic factors by these three diseases could help identify new asthma loci.

**Methods:**

GWAS-nominated LC and COPD loci were selected among studies performed in Caucasian cohorts using the GWAS Catalog. Genetic analyses were carried out for these loci in the Saguenay–Lac-Saint-Jean (SLSJ) asthma familial cohort and then replicated in two independent cohorts (the Canadian Cohort Obstructive Lung Disease [CanCOLD] and the Epidemiological Study of the Genetics and Environment of Asthma [EGEA]).

**Results:**

Analyses in the SLSJ cohort identified 2851 and 4702 genetic variants to be replicated in the CanCOLD and EGEA cohorts for LC and COPD loci respectively. Replication and meta-analyses allowed the association of one new locus with asthma, 2p24.3, from COPD studies. None was associated from LC studies reported.

**Conclusions:**

The approach used in this study contributed to better understand the heritability of asthma with shared genetic backgrounds of respiratory diseases.

**Supplementary Information:**

The online version contains supplementary material available at 10.1186/s12890-022-01890-7.

## Background

The World Health Organization (WHO; www.who.int) has stated that noncommunicable diseases are the world’s biggest killers, with 36 million deaths globally on an annual basis. Cancers account for 21% of these, particularly lung cancer (LC) which is the leading cause of death. Chronic respiratory diseases represent 12% of deaths, and include asthma and chronic obstructive pulmonary disease (COPD). These three noncommunicable diseases are characterized by the combination of complex mechanisms underlying and genetic predispositions, with asthma having the highest calculated heritability (55–90%) [1]. Efforts in the last decades have defined multiple genetic factors, with more than 100 loci linked to asthma in genome-wide association studies (GWAS) [1]. However, they explain only a small part of this heritability; undiscovered genes may reside in genetic loci common between asthma and other respiratory diseases such as LC and COPD.

Meta-analyses have shown an increased risk of developing LC for asthmatic individuals [2, 3]. However, it was recently demonstrated that these associations were probably biased by other respiratory or allergic comorbidities. In fact, when analyzing data from individuals with asthma only, an inverse relationship was found [3], suggesting a protection against LC for asthmatics. Links between asthma and COPD have also been studied, both diseases being frequent comorbidities with up to 55% with COPD having the other as well [4]. In recent years, an asthma-COPD overlap (ACO) syndrome has been described. However, as these two diseases are each characterized by complex interactions of environmental and genetic factors, it is now recognized that ACO is as heterogeneous as the two others and do not correspond to a syndrome definition [5]. Nonetheless, a genetic correlation of 0.38 has been observed between asthma and COPD [6].

In this study, we hypothesized that genetic factors shared by LC or COPD and asthma could help identify new loci of interest for the latter and thus contribute in defining the missing heritability of this complex trait.

## Methods

**Fig. 1:**
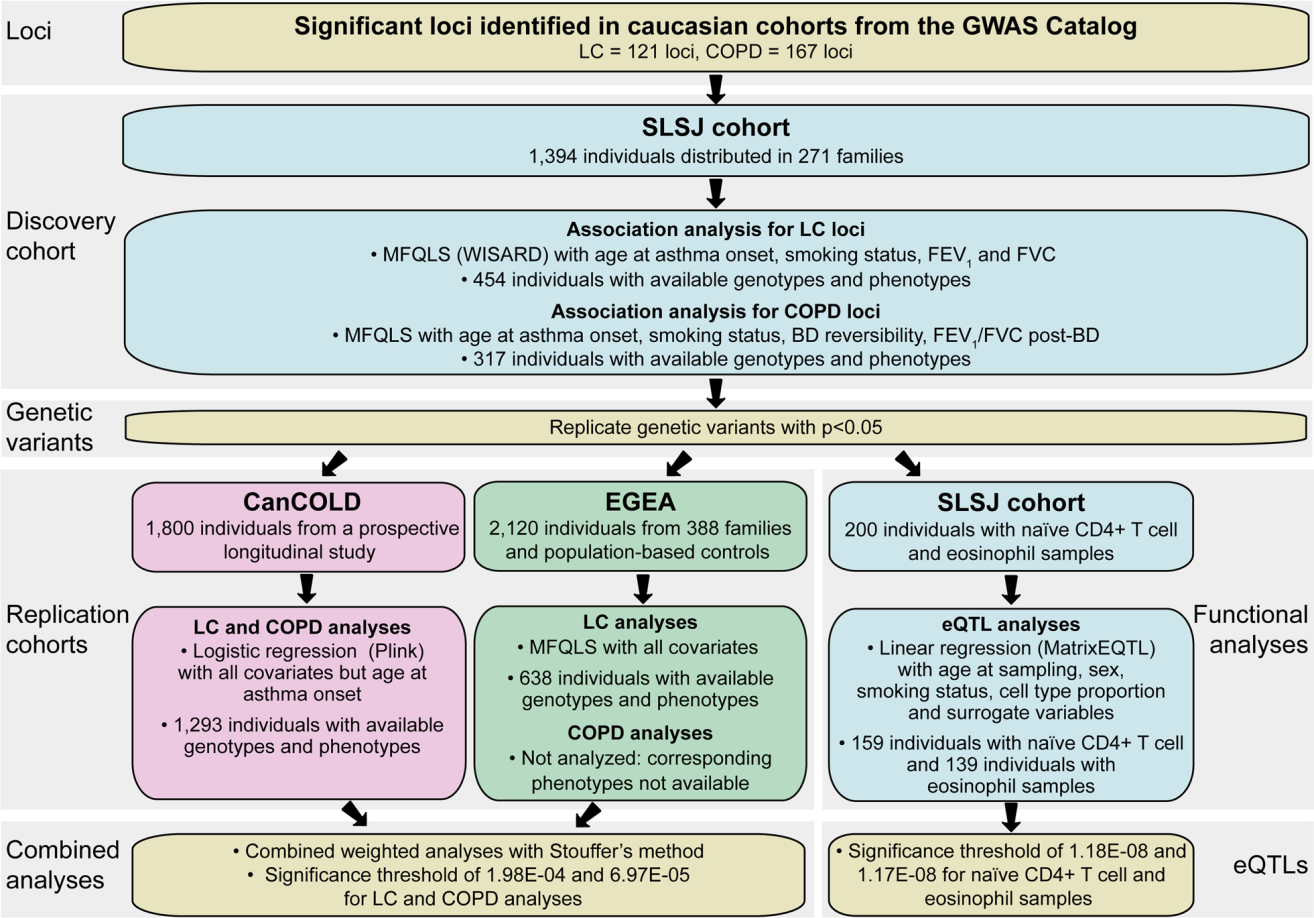
Study design. This figure shows the schematic view of the study design, describing each analysis steps performed in the discovery cohort and the replication ones as well as for functional analyses. BD = bronchodilator, CanCOLD = Canadian Cohort Obstructive Lung Disease, COPD = chronic obstructive lung disease, eQTL = expression quantitative trait locus, EGEA = The French Epidemiological study on the Genetics and Environment of Asthma, FVC = forced vital capacity, FEV_1_ = forced expiratory volume in 1 second, LC = lung cancer, SLSJ cohort = Saguenay–Lac-Saint-Jean asthma familial cohort

A schematic view of the study design is presented in Fig. 1 with short description of cohorts, analyses performed including covariates, and significance thresholds.

### Discovery cohort

The Saguenay–Lac-Saint-Jean asthma familial cohort (SLSJ cohort) [7] comprises 271 families ascertained through one proband (offspring) with allergic asthma for a total of 1394 individuals, among which 1214 have phenotypes and genotypes available. Genome-wide methylation and expression data are also accessible for 215 of these individuals. The general and respiratory health of all were evaluated using a standardized questionnaire (including questions about smoking status, age at asthma onset, etc.) as well as allergy testing and pulmonary function tests according to the American Thoracic Society guidelines (as the forced expiratory volume in 1 second [FEV_1_], the forced vital capacity [FVC], the FEV_1_/FVC ratio post bronchodilator [BD] treatment with inhalation of a 200 ng salbutamol dose, the BD reversibility [FEV_1_ post-BD minus FEV_1_ pre-BD], and the methacholine challenge [PC_20_]) [8, 9]. Participants were considered asthmatic if: (1) they had a reported history of asthma (validated by a physician), or (2) they presented asthma-related symptoms and positive PC_20_ (< 8 mg/ml) on recruitment. All participants (or kin or guardians of minors) gave informed written consent and the study was approved by the *Centre intégré universitaire de santé et de services sociaux du Saguenay–Lac-Saint-Jean* ethics committee (project #0002-001, 08-11-2005).

### Replication cohorts

Each site of both replication cohorts obtained local Research Ethics Board approval for the study, and each participant gave signed informed consent. The Canadian Cohort Obstructive Lung Disease (CanCOLD) [10] is a prospective longitudinal study comprising 1800 individuals with assessment at the time of recruitment, 18 months, 3 years and so on. It involves two balanced subsets of COPD, mild and moderate-to-severe COPD, defined based on Global Initiative for Chronic Obstructive Lung Disease (GOLD) classification (https://goldcopd.org/) as well as many subsets of non-COPD individuals with normal post-bronchodilator spirometry including ever smokers, for those at risk, and never smokers, for healthy controls matching for sex and age. Asthma phenotype, as well as respiratory measures, is similar to the SLSJ cohort. Age at asthma onset is unavailable for this cohort.

The French Epidemiological study on the Genetics and Environment of Asthma (EGEA) [11] combines a family-based study on this condition and a case–control study consisting of three surveys over 20 years, from 1991 to 2013 (see https://egeanet.vjf.inserm.fr/index.php/en/ for details). The whole study includes 388 asthmatic probands and their 1317 first-degree relatives additional to 415 population-based controls (total of 2120 subjects). All EGEA participants have extensive phenotypic characterization through use of standardized questionnaires as well as physiological and biological tests, and data on many lifestyle and environmental factors. However, values for BD reversibility and FEV_1_/FVC ratio from a post-BD treatment were not available for the EGEA study.

### Genetic data

Genetic data for the SLSJ cohort were collected from Illumina Human610-Quad BeadChip. A complete description of the quality control filtering and the imputation process is available in Madore et al. [12]. Briefly, the pre-phasing step has been performed with the Shapeit2 software [13] for imputation in turn by the Impute2 software [14] with samples from the 1000 Genomes Project (phase 3) [15] and the UK10K as reference. After quality control filtering, imputed genotypes were accessible for 1198 samples and according to availability of phenotypes and covariates, analyses have been performed on genotypes from 454 individuals for lung cancer loci and 317 for COPD loci.

Genotypes for CanCOLD were obtained from the Infinium Global Screening Array-24 v2.0 BeadChip. Imputations were carried out using the same quality control criteria, tools and reference samples as the SLSJ cohort. After quality control filtering and according to availability of phenotypes and covariates, analyses were made on genotypes from 1293 individuals for LC and COPD susceptibility loci.

Genetic data were available for 1904 EGEA participants (Illumina Human610-Quad BeadChip). Imputations were carried out using the Haplotype Reference Consortium panel (> 20 million SNPs). Stringent quality control was applied before and after the imputation, and imputed genotypes with posterior probabilities < 0.9 were set to missing. After quality control filtering and according to availability of phenotypes and covariates, analyses for lung cancer susceptibility loci were made on genotypes from 638 offsprings.

### Genetic analyses

On November 19, 2019, GWAS Catalog (https://www.ebi.ac.uk/gwas/) was used to list all GWAS nominated loci associated with LC and COPD on Caucasian cohorts. The keywords “familial lung cancer”, “small cell carcinoma”, “non-small cell carcinoma (adenocarcinoma and squamous cell carcinoma)” and “pulmonary neuroendocrine tumor” were typed to search for LC loci and “chronic obstructive pulmonary disease” was for COPD loci. GWAS that are addressing treatment response were excluded. Regions of ± 100 kb around GWAS-associated genetic variants were used for analysis in the  SLSJ cohort [7].

We carried out the MFQLS test implemented in the Workbench for Integrated Superfast Association study with Related Data (WISARD) toolkit [16, 17] in the SLSJ samples to identify single nucleotide polymorphisms (SNPs) or insertions/deletions associated with asthma at a *p* value < 0.05. MFQLS is a quasi-likelihood method of estimation extended from the Cochran-Armitage’s one and suited for familial cohorts considering kinship coefficients [16, 17]. From these, significance thresholds for replication were calculated with Nyholt’s method [18], in accordance with the number of independent genetic variants in LC and COPD susceptibility loci and were set at 1.98E−04 (259 variants) and 6.97E−05 (736 variants), respectively. For LC, analyses were made with the asthma phenotype using age at asthma onset, smoking status (smokers [smokers and ex-smokers who have stopped for less than a year], ex-smoker [if stopped for at least one year] and non-smoker), FEV_1_ and FVC in percentages of predicted value as covariates. For COPD, they were with the asthma phenotype and age at asthma onset, smoking status, BD reversibility and FEV_1_/FVC ratio from a post-BD treatment used as covariates.

Analyses in the CanCOLD cohort for lung cancer  LC and COPD susceptibility loci were made using the logistic function in Plink1.9 with all covariates excepts age at asthma onset, which was unavailable. As for the EGEA study, they have been performed with the MFQLS test for lung cancer loci only, as BD reversibility and FEV_1_/FVC ratio from a post-BD were not available here for associations with COPD loci.

Results from the SLSJ discovery cohort and both replication ones were combined using the Stouffer’s approach implemented in the METAL software considering the weight of each cohort (http://csg.sph.umich.edu/abecasis/metal/) [19]. Direction of associations were not considered. As for Haploview, it was used to delimit the haplotype blocks in the regions associated with asthma using the analyses in combination with an algorithm taken from Gabriel et al. [20, 21].

### Isolation of naïve CD4^+^ T cells as well as eosinophils, and RNA sequencing

To look at possible expression quantitative trait loci (eQTLs) between associated SNPs and gene expression in immune cell types involved in asthma pathophysiology, transcriptome data of naïve CD4^+^ T cells (units with CD3^+^, CD4^+^, CD45RA^+^, and CD45RO^−^ markers) and eosinophils from 215 individuals of the SLSJ cohort were used [12]. Such analyses may help better understand possible functional impact of associated genetic variants. Those cells were isolated from 200 ml of blood samples and gene expression counts were extracted from sequencing data of 500 ng RNA samples. For details on the method, please see Madore et al. [12]. After quality control filtering and given availability of all covariates included in statistical models, transcriptomic data were accessible for 159 naïve CD4^+^ T cell samples and 139 eosinophil samples.

### eQTL analyses

eQTLs analyses have been performed using the package MatrixEQTL in R [22] for gene expression counts with age at sampling, sex, smoking status, proportion of naïve CD4^+^ T cells or eosinophils, according to which sample types were used for analyses, and surrogate variables as covariates. Proportion of cell types were calculated with the methylome data collected from the same samples and the Houseman method [23] implemented in R package RnBeads [24] as previously described [12]. Surrogate variables were using the R package sva [25] and were added to the analysis model in order to take into account hidden confounders and relatedness between samples. The significance threshold was calculated with Nyholt’s method [18], in accordance with the number of independent genetic variants in LC and COPD susceptibility loci in a respective manner, multiplied by that of genes expressed by each cell type. Those thresholds were set at 1.18E−08 and 1.17E−08 for LC loci when analyzing naïve CD4^+^ T cells (259 independent genetic variants × 16,324 genes) and eosinophils (259 × 16,449), respectively, and at 4.16E−09 and 4.13E−09 for COPD loci for analyses in naïve CD4^+^ T cells (736 × 16,324) and eosinophils (736 × 16,449), respectively. Analyses were run for cis-eQTLs (maximum distance between gene and genetic variant ≤ 100 kb) and trans-eQTLs (distance between gene and genetic variant > 100 kb) separately.

Finally, the eQTL Catalogue (https://www.ebi.ac.uk/eqtl/), that includes data from several studies including the GTEx Portal, was used to look at eQTLs between associated SNPs and all tissue types.

## Results

According to the GWAS Catalogue, 121 LC loci and 167 COPD loci have been selected for analyses (Additional file 1: Tables S1 and S2), representing 60,379 and 85,776 genetic variants in the SLSJ cohort respectively. Analyses have been performed in this cohort using the MFQLS method. Among the genetic variants tested, 2956 and 4893 for LC and COPD loci respectively had *p* values below or equal to 0.05 in SLSJ. Of these, 2851 and 4702 were available in at least one of two independent cohorts of Caucasian origins (CanCOLD or EGEA cohorts) and were selected for replication (Additional file 1: Tables S3 and S4). LC variants were tested for replication in both cohorts. A logistic model was used for the CanCOLD [10] which has a case–control design, and the MFQLS method was for the EGEA which has a familial design [11]. No variants were found significantly associated in the meta-analysis.

**Fig. 2:**
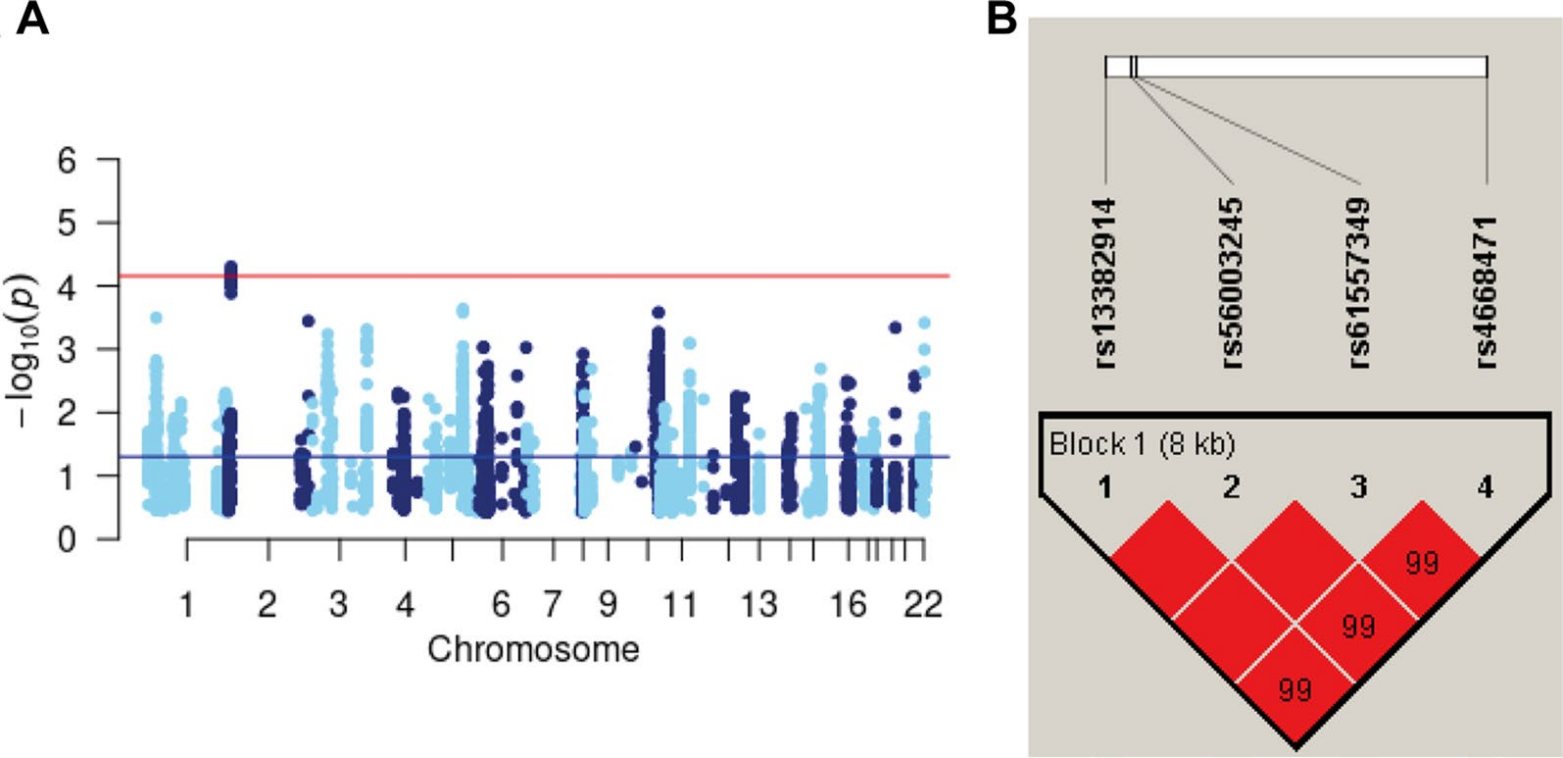
Haplotype blocks for the asthma-associated locus. This figure shows the Manhattan plot **a** for the meta-analysis including the Saguenay–Lac-Saint-Jean asthma familial cohort (SLSJ cohort) and the Canadian Cohort Obstructive Lung Disease (CanCOLD) for the 2p24.3 COPD locus, and **b** haplotype blocks formed by the 4 SNPs associated with asthma in the 2p24.3 COPD locus using genotypes from the SLSJ cohort  (done with Haploview software). The color of squares illustrates the strength of pairwise linkage disequilibrium (LD) values on a red and white scale where red indicates perfect LD (r^2^ = 1.00) and white perfect equilibrium (r^2^ = 0)

COPD genetic variants were only replicated in the CanCOLD [10] cohort, according to covariates availability. Among these, four were found significantly associated with asthma in the meta-analysis including both cohorts (*p* < 6.97E−05) (Table 1, Fig. 2). These (2p24.3 locus, chr2:15,969,655–15,977,866) are 63.5 kb downstream of the COPD-associated variant in the literature (rs10929386, not evaluated in the SLSJ cohort [best r^2^ with the nearest at < 0.01]) [26] and form a unique haplotype block (Fig. 2B). These variants are 10.3 kb upstream the MYCN proto-oncogene (*MYCN*) gene’s transcription start site. According to data from the SLSJ cohort, this haplotype block includes 34 genetic variants, all significant in the SLSJ cohort, among which 4 were genotyped and 30 were imputed (Additional file 1: Table S3). Looking at ENCODE and ORegAnno databases of regulatory elements, the haplotype block of 34 genetic variants spans several regulatory features. A DNAse 1 hypersensitivity site with a score of 1000/1000 was found at position chr2:15,980,166–15,980,675, a H3K27me3 modification site with a score of 789 out of 1000 at position chr2:15,975,986–15,976,193 and two transcription factor binding sites for E2F1 at position chr2:15,969,690–15,970,109 and JUND at position chr2:15,975,707–15,975,721.

**Table 1:** Significant associations of chronic obstructive pulmonary disease loci with asthma in the combined analysis of two Caucasian cohorts

CHR	SNP^a^	HGVS name	Genotyped or imputed	MAF SLSJ/UCSC^b^	Nearest gene	r^2c^	SLSJ cohort		CanCOLD		Combined (SLSJ, CanCOLD)	
							Effect allele	*P*	Effect allele	*P*	Z-score	*P*
2	rs13382914	g.15969655C>T	Imputed	0.23/0.42	111,028 bp before *MYCN*	0.995	T	0.011	T	0.001	4.014	5.98E−05
	rs56003245	g.15970221T>A	Imputed	0.23/0.43	110,462 bp before *MYCN*	0.995	A	0.018	A	0.001	3.990	6.62E−05
	rs61557349	g.15970339A>G	Imputed	0.23/0.42	110,344 bp before *MYCN*	0.995	G	0.018	G	0.001	− 3.990	6.62E−05
	rs4668471	g.15977866A>T	Imputed	0.23/0.53	102,817 bp before *MYCN*	1.000	T	0.009	T	0.001	− 4.056	4.99E−05

Chr, chromosome; CanCOLD, Canadian Cohort Obstructive Lung Disease; HGVS, human genome variation society; MAF, minor allele frequency; SLSJ cohort, Saguenay–Lac-Saint-Jean asthma familial cohort

^a^SNPs identified using data from dbSNP151

^b^HGVS names and distance from genes’ transcription start sites calculated from human genome version 19 (hg19, GRCh37). †MAF for the SLSJ cohort and the 1000 Genomes project available from UCSC Genome Browser (https://genome.ucsc.edu)

^c^R square between the most significant genetic variant and the three other ones

eQTL analyses were performed between SNPs of all GWAS-nominated loci for LC and COPD in naïve CD4^+^ T cell and eosinophil samples from the SLSJ cohort. Following analyses of LC loci, a total of 622 and 1682 significant cis- and trans-eQTLs were found for naïve CD4^+^ T cell samples respectively and a total of 204 and 1334 cis- and trans-eQTLs were found for eosinophil samples respectively (Additional file 1: Table S4). Following analyses of COPD loci, a total of 148 and 384 significant cis- and trans-eQTLs were found for naïve CD4^+^ T cell samples respectively and a total of 0 and 355 cis- and trans-eQTLs were found for eosinophil samples respectively. None were identified for the four associated SNP (Additional file 1: Table S5). No documented eQTLs were found in eQTL Catalogue for associated SNPs.

## Discussion

Studying GWAS-nominated loci of LC and COPD, this study allowed to identify one new asthma locus (2p24.3 locus near *MYCN* gene). This gene encodes a transcription factor normally involved in development of multiple organs including the nervous system and lungs, and aberrantly functions as a known oncogene, predominantly in neuroblastoma [27]. Results for LC and COPD loci are in line with the insufficient evidence observed for common biological background between asthma and LC, compared to the known ties between asthma and COPD, as much for the high proportion of individuals affected by both diseases [4], as for the genetic correlation between them [6]. Even considering this correlation, a previous study of the UK Biobank team including 28,628 individuals with asthma and 9266 individuals with COPD didn’t find any common genes between results from gene-based association tests for these two diseases [28]. This could be due to the high phenotypic heterogeneity of both diseases and may explain why only one common locus was found in this study.

Neither the gene *MYCN* proximal to the genetic variants associated with asthma in our study, nor its respective locus, have previously been associated with asthma or allergy in the literature. Moreover, considering all tissues available, no documented eQTLs were found in the eQTL Catalogue for associated SNPs, nor in naïve CD4^+^ T cells or eosinophil samples from the SLSJ cohort. eQTLS could have helped explain association of the four SNPs with asthma through a possible functional link with proximal or distal genes. However, GTEx databases do not include all isolated cell types as circulating immune cells and thus, eQTLs may be found in others than the ones tested in the SLSJ cohort or listed in GTEx databases and the eQTL Catalogue. According to the ENCODE project, the haplotype block identified in the SLSJ cohort spans several regulatory features including binding sites for the transcription factors E2F1 and JUND. Gene expression studies have linked *E2F1* expression levels with childhood and allergic asthma [29, 30] underlying possible implications of this new associated locus in asthma.

The analyses performed on eosinophil and naïve CD4^+^ T cell samples from the SLSJ cohort have identified several eQTLs for the genetic variants associated with asthma in the discovery cohort. Even though this study did not prove any link for these eQTLs with asthma, as the association of their respective variants has not been replicated successfully, these results are a contribution to the general knowledge on their integration with gene expression in human samples of isolated immune cells, including a rarely studied one, the eosinophils.

## Conclusions

This study was based on the hypothesis that genetic backgrounds common between asthma and other respiratory diseases such as LC and COPD will lead to the identification of new heritable loci of interest for this disease. Indeed, our analyses using three cohorts have shown robust associations for a new locus at 2p24.3. Our results suggest that this one warrant further studies in additional independent cohorts as well as functional genetic analyses.

## Supplementary Information

**Additional file 1: Table S1.** Loci associated with lung cancer (LC) in GWAS performed in Caucasian cohorts. **Table S2.** Loci associated with chronic obstructive pulmonary disease in GWAS performed in Caucasian cohorts. **Table S3.** Association results for the chronic obstructive pulmonary disease loci in the Saguenay–Lac-Saint-Jean asthma familial cohort for genetic variants forming an haplotypic block with significant variants of the 2p24.3 locus in the combined analysis. **Table S4.** Associations with *p* < 0.05 for the lung cancer loci in the Saguenay–Lac-Saint-Jean asthma familial cohort, replication in two other cohorts and expression quantitative trait loci analyses in two immune cell types. **Table S5**. Associations with *p* < 0.05 for the chronic obstructive pulmonary disease loci in the Saguenay–Lac-Saint-Jean asthma familial cohort (SLSJ cohort), replication in another cohort and expression quantitative trait loci analyses in two immune cell types. Additional files with all GWAS loci identified in the literature for the lung cancer and chronic obstructive pulmonary diseases publications in the GWAS Catalogue, complete description of the 2p24.3 haplotype block and all significant associations found in the SLSJ cohort as well as eQTLs.

## Data Availability

Datasets used or analyzed during the current study are available from the corresponding author on reasonable request. The data are not publicly available due to ethical reasons.

## Abbreviations

ACO: Asthma-COPD overlap
BD: Bronchodilator
CanCOLD: The Canadian Cohort Obstructive Lung Disease
COPD: Chronic obstructive pulmonary disease
EGEA: The French Epidemiological study on the Genetics and Environment of Asthma
eQTL: Expression quantitative trait locus
FEV_1_: Forced expiratory volume in 1 second
FVC: Forced vital capacity
GWAS: Genome-wide association study
LC: Lung cancer
*MYCN*: MYCN proto-oncogene gene
PC_20_: Methacholine challenge that induce a 20% fall in FEV_1_
SLSJ: Saguenay–Lac-Saint-Jean
WHO: World Health Organization
WISARD: Workbench for Integrated Superfast Association study with Related Data
